# Subcutaneous natalizumab administration in relapsing–remitting multiple sclerosis: results of EASIER 2 study

**DOI:** 10.1007/s00415-026-13977-w

**Published:** 2026-07-10

**Authors:** Massimo Filippi, Luigi M. E. Grimaldi, Vincenzo Brescia Morra, Antonella Conte, Cinzia Cordioli, Rocco Totaro, Giacomo Lus, Augusto Rini, Fabiana Marinelli, Paola Valentino, Paola Perini, Girolama Alessandra Marfia, Mariarosaria Valente, Simona Malucchi, Chiara Zanetta, Lorenzo Pradelli, Daria Perini, Laura Santoni, EASIER 2 study working Group

**Affiliations:** 1 Neurology Unit, Neurorehabilitation Unit, Neurophysiology Service, and Neuroimaging Research Unit, Division of Neuroscience, IRCCS San Raffaele Scientific Institute, Via Olgettina, 60, 20132 Milan, Italy; 2 Vita-Salute San Raffaele University, Milan, Italy; 3 Neurology Unit, Multiple Sclerosis Center, Fondazione Istituto G. Giglio, Cefalù, Italy; 4 UniCamillus–Saint Camillus International University of Health Sciences, Rome, Italy; 5 Multiple Sclerosis Clinical Care and Research Center, Department of Neuroscience (NSRO), Federico II University, Naples, Italy; 6 Department of Human Neurosciences, Sapienza University of Rome, Rome, Italy; 7 IRCCS Neuromed, Pozzilli, IS Italy; 8 Multiple Sclerosis Center, ASST Spedali Civili Di Brescia, Montichiari Hospital, Brescia, Italy; 9 Demyelinating Disease Center, Department of Neurology, San Salvatore Hospital, L’Aquila, Italy; 10 Multiple Sclerosis Center, UOC II Neurology, Department of Advanced Medical and Surgical Sciences, University of Campania “L. Vanvitelli”, Naples, Italy; 11 Multiple Sclerosis Center, Division of Neurology, A. Perrino Hospital, Brindisi, Italy; 12 MS Center—Neurology Unit, F. Spaziani Hospital, Frosinone, FR Italy; 13 Department of Neurology, Magna Graecia University of Catanzaro, Catanzaro, Italy; 14 Multiple Sclerosis Centre, University Hospital of Padua, Padua, Italy; 15 Multiple Sclerosis Clinical and Research Unit, Department of Systems Medicine, Tor Vergata University, Rome, Italy; 16 Clinical Neurology, Department of Medicine (DMED), University of Udine, Udine, Italy; 17 SCDO Neurologia—CReSM, AOU San Luigi Gonzaga, Orbassano, Turino, Italy; 18 AdRes, Turin, Italy; 19 Biogen Italia, Milan, Italy

**Keywords:** Multiple sclerosis, Natalizumab, Subcutaneous administration, Quality of life, Health economics

## Abstract

**Introduction:**

EASIER 2 is a multicenter, observational, cross-sectional study conducted in 14 Italian multiple sclerosis (MS) centers to evaluate patient and healthcare professional (HCP) time, healthcare resource consumption, costs, and patient quality of life (QoL) associated with subcutaneous (SC) administration of natalizumab compared to intravenous (IV) administration as measured in the earlier EASIER study.

**Methods:**

The study included: (1) time-and-motion analysis using a mobile application to collect real-world data during SC administration; (2) structured HCP questionnaire assessing organizational impacts; (3) patient survey capturing experience, preferences, and QoL. The comparison between SC and IV was conducted using a random-intercept regression model adjusted for patient history.

**Results:**

A total of 265 SC procedures were analyzed. Compared to IV administration, SC reduced patient center time by 77%, HCP active time by 35%, and chair time by 74%. Hospital costs per administration decreased by 60%, with a reduction of about 33 and 14% from the perspectives of the Society and patient, respectively. Among patients, 86% preferred SC administration, mainly due to shorter center time, less physical discomfort, and lower emotional burden. QoL scores (scale 0–100) were higher on SC days (mean 70.4) than IV days (mean 56.9) administration, corresponding to a 13-point improvement (*p* < 0.001). All HCPs reported a favorable opinion on SC administration, with 82% expecting simplifications in MS center organization and shorter patient waiting lists.

**Conclusions:**

SC natalizumab administration significantly reduces dispensation procedure times, healthcare resource consumption, and associated costs, while improving patient experience and QoL.

**Supplementary Information:**

The online version contains supplementary material available at 10.1007/s00415-026-13977-w.

## Introduction

Multiple sclerosis (MS) is a chronic and disabling disease that primarily affects young adults in their working years [1, 2]. As the disease progresses, symptoms gradually worsen, including fatigue, reduced mobility, low mood, disability, and cognitive decline [2]. Consequently, MS has a direct impact on patients’ functionality and quality of life (QoL), leading to increased use of healthcare and non-healthcare resources, as well as a reduction in work capacity and productivity [2]. The chronic and progressive nature of MS, along with the young age at diagnosis, result in a significant burden on patients and their families, society, and the National Health Service (NHS) [2].

Natalizumab is a monoclonal antibody used to treat patients with highly active relapsing–remitting MS (RRMS) [3]. It controls and reduces inflammatory activity of MS, decreases axonal damage, and delays the progression of motor and cognitive disability, thereby having a substantial long-term impact on disease progression [4, 5].

The therapeutic regimen requires hospital administration, either intravenously (IV) via slow infusion lasting at least 60 min or subcutaneously (SC) through two injections administered within 30 min. Both administration methods are followed by a monitoring phase according to number and type of administration [6, 7].

It is reasonable to expect that these two administration routes lead to different levels of healthcare resource utilization, including healthcare professionals’ working time and infusion chair occupancy. In particular, the SC formulation has the potential to significantly reduce healthcare resource consumption, as observed in the previous studies conducted in other therapeutic settings [8–12].

In 2023, before the availability of the new SC formulation of natalizumab in routine clinical practice in Italy, the EASIER study was published [13], in which the administration times for IV natalizumab in RRMS patients were measured using a time-and-motion methodology and compared with estimated SC administration times and resources consumption as reported by healthcare professionals (HCPs). The measurement of over 400 procedures revealed that patients spent an average of 152 min at the center per each IV infusion. The active working time required by HCPs was 29 min per IV administration. With the adoption of the SC route, HCPs estimated a 50% reduction in patient procedure time and a 55% decrease in their active working time. This would correspond to a 63% decrease in costs incurred by the MS center for each natalizumab administration.

After the introduction and use of SC natalizumab in Italy, we chose to conduct the present analysis by employing the same time-and-motion methodology to directly measure the administration times for SC natalizumab and compared them with the previously measured IV administration times from the EASIER study [13]. The objective of this study, called “the EASIER 2 study”, was to assess the economic impact of adopting SC natalizumab compared to IV natalizumab in the Italian healthcare setting, considering perspectives from multiple stakeholders, including MS centers, patients, and society as a whole. In particular, preparation and administration times, healthcare resource utilization, MS center organization, as well as patient time, costs, and patient QoL were investigated.

## Methods

### Study design

The EASIER 2 study was a multicenter, observational, non-pharmacological, cross-sectional study conducted in the period March 2024–July 2024 in 14 Italian MS centers. It was made by three parts:

- part 1: evaluation of healthcare resources associated with SC administration using the time-and-motion micro-costing methodology, i.e., direct observation of activities, time, and materials used in SC administration conducted by HCPs as part of routine clinical practice by means of a dedicated App (EASIER App, as in the EASIER study [13]). The collected data were then monetized based on unit costs obtained from the hospital management center, also through the same App;
- part 2: HCP preferences and impact on MS center organization. Direct data collection through a structured survey conducted via a web-based questionnaire administered to HCPs involved in part 1;
- part 3: patient time, satisfaction, and preferences. Data were gathered through a web-based questionnaire administered to patients and included collection of time required for SC natalizumab administration by patients and, if applicable, caregivers, including time for transportation, formal and informal assistance, and work absence, together with preferred route of administration, and impact on QoL. These data were then monetized to estimate the direct, indirect, and intangible costs associated with SC administration for patients and their families.

### Participating centers

The centers which participated in the study were: IRCCS San Raffaele Scientific Institute (Milan), Montichiari Hospital (Brescia), AOU S. Luigi Gonzaga University Hospital (Orbassano, TO), Department of Medicine (DMED) University of Udine (Udine), San Salvatore Hospital (L’Aquila), Department of Human Neurosciences, Sapienza University of Rome (Rome), Multiple Sclerosis Clinical Care and Research Center, Federico II University (Naples), Multiple Sclerosis Center, Fondazione Istituto G. Giglio (Cefalù, PA), Department of Neurology, Magna Graecia University of Catanzaro (Catanzaro), Multiple Sclerosis Clinical and Research Unit, Tor Vergata University (Rome), Multiple Sclerosis Center, Fabrizio Spaziani Hospital (Frosinone), Multiple Sclerosis Centre, University Hospital of Padua (Padua), Multiple Sclerosis Center, A. Perrino Hospital (Brindisi), Multiple Sclerosis Center, Second University of Naples (Naples).

### Population

The EASIER 2 study recruited adult patients (≥ 18 years) diagnosed with RRMS according to McDonald criteria who attended one of the above-mentioned MS centers to receive SC natalizumab administration according to the approved indication as part of previously scheduled routine clinical practice. Patients were included if they showed willingness to participate in the study and if they signed the informed consent form.

### Endpoints

The primary endpoint of the study was the comparison of the mean cost of IV and SC natalizumab administration regimens for the treatment of patients with RRMS from the perspective of the MS center. The total cost was detailed as follows: healthcare resources consumed (medications and consumables), active working time spent by the HCPs, and use of durable equipment.

The secondary endpoints were:

- comparison between SC and IV administration in terms of active working time of HCPs (total and broken down by professional role and activity);
- comparison between SC and IV administration in terms of patient length of stay at the MS center for administration and chair time;
- mean cost borne by the patient and society under the SC administration regimen, both total and by component (healthcare and non-healthcare direct costs and indirect costs);
- intangible cost (related to the perceived loss of QoL) for patients receiving SC treatment;
- mean cost of SC administration from a societal perspective.

### Data collection

Patients were invited to participate in the study during a routine visit to the MS center for their scheduled SC natalizumab administration. Clinicians or nurses introduced the study, outlining its objectives and design, and provided patients with written information. Those willing to participate gave their informed consent, allowing the processing of their personal data.

In part 1, each participating center was required to measure the time and resources’ utilization for a series of SC natalizumab administrations (≤ 20) as part of routine clinical practice. A protocol amendment allowed centers to recruit a higher number of patients; thus, 5 centers exceeded 20 patient recruitment. HCPs conducted these measurements using the EASIER App, which enabled them to select a specific procedure and task to be recorded during natalizumab SC administration. Each measurement was captured using start/stop buttons, whereas resource usage was documented through a selection of predefined items within the App. The EASIER App supported simultaneous data collection by multiple HCPs and allowed a single or multiple operators to track concurrent procedures. It was installed on the mobile devices of the participating researchers to facilitate real-time data entry. The time and resources related to SC natalizumab administration collected during part 1 were compared with those related to IV natalizumab administration gathered during the EASIER study [13] using the same App.

In part 2, HCP preferences and impact on MS center organization about SC administration (such as rooms designated for drug administration, number of administration chairs, number of HCPs on duty simultaneously, and number of patients attended to simultaneously by each HCP) were investigated using a questionnaire called “How much EASIER 2?”. It was available online, with printed copies provided upon request. All collected data were entered into the centralized EASIER study database. Printed responses were scanned and manually input into the system. Each center was required to recruit at least one representative for each professional role involved (e.g., one physician, one nurse, etc.).

In part 3, the assessment of costs and QoL for patients receiving SC natalizumab was conducted through a questionnaire called “EASIER for you 2?”, which was made available online, with printed copies provided upon patient request, and could be completed independently by enrolled patients or with assistance from a caregiver or HCP. All collected data were stored in the centralized EASIER study database. As part of the questionnaire, patients were asked to assess their QoL using the Visual Analog Scale (VAS), which ranges from 0 (lowest possible QoL) to 100 (highest possible QoL). Additional exploratory analyses were conducted to examine patient preferences regarding the different administration routes of natalizumab (SC or IV). Data were also collected about hours of lost patient productivity and the need for assistance from caregivers (relatives/friends or paid workers).

Since the study followed a cross-sectional design, data were collected only once, with no follow-up planned. The resulting sample size was considered sufficient to perform a health economics analysis.

### Data management

As detailed in Online Resource 1, to facilitate data collection using the EASIER App, the administration procedure was divided into macro-tasks, which afterward were grouped for subsequent analysis. In cases where time recordings were missing due to errors during data entry, they were excluded from the analysis.

### Statistical analyses

Categorical variables were presented as absolute and relative frequencies (percentages), while continuous variables were summarized using the mean, standard deviation, median, and range.

In addition to the descriptive statistics mentioned above, SC procedure times (HCP time, patient time, and chair time) were estimated using a random-intercept regression model to account for potential organizational differences among participating centers. Variables were adjusted for the number of previous infusions (both IV and SC) to account for the possible confounding effect of the HCP’s prior experience with the patient (execution times may be longer for newly treated patients, as HCPs might need to explain steps or answer questions). Briefly, the model used in this analysis is expressed as follows:

$$Y = \alpha_{center} + \beta_{IV} \times \# IV \inf usions + \beta_{SC} \times \# SC \inf usions + \varepsilon$$

where *Y* is the dependent variable (HCP/patient/chair time), *α*_*center*_ represents the estimate of *Y* adjusted for prior infusions, *β*_*IV*_ and *β*_*SC*_ represent the increase in *Y* for each additional previous IV or SC infusion, respectively, and *ε* is the error term for observed data (assumed to be normally distributed with a mean of zero and same variance across measurements). In the random-intercept model, the *α*_*center*_ parameter follows a normal distribution centered around a mean value *α*, with variance *τ*_*center*_ describing variability in *Y* across the different centers.

Finally, the data collected in the previous EASIER study on IV natalizumab administration times [13] were compared with those collected in the present study using a similar random-intercept regression model. In this case, a “formulation” variable was added to estimate the potential reduction in time (HCP/patient/chair) associated with switching to the SC formulation. To minimize selection bias due to differences in participating centers between the two studies, this analysis was limited to data collected from centers that participated in both the EASIER and EASIER 2 studies.

The QoL values reported for the three types of days were compared using a linear regression model adjusted for age, gender, previous experience with IV natalizumab, previous experience with SC natalizumab, and employment status (paid work: yes vs no). In scenario analyses, the model was also adjusted for EDSS score, which was not included in the base case due to missing data in over 20% of the sample. EDSS was included both as continuous and dichotomous variable (EDSS < 4 vs EDSS ≥ 4).

Finally, a possible interaction between disease severity and the type of day on which QoL was assessed was tested by using the interaction test.

Exploratory analyses on center-level characteristics (e.g., available stations, nurses/patient, etc.) were conducted to identify possible factors of inter-center variability.

### Health economics analyses

#### MS center perspective

Each healthcare resource consumed was valued using a unit cost, either collected within the EASIER App or derived from national-level literature sources [14, 15].

The labor cost of HCPs was valued according to the opportunity-cost principle and based on the national average wages reported by ISTAT, i.e., 67 €h and 27 €/h for physicians and nurses, respectively [14].

The use of durable medical equipment was assigned a cost that varied by center, calculated as the ratio between the hourly cost of an infusion room (€ 4/h) [15] and the number of infusion chairs reported by each center in the “How much EASIER 2?” questionnaire.

The consumption of materials required for administration was valued using unit costs collected during the study.

To determine the total cost, in line with best practice for activity-based costing [12], hospital general costs must also be considered. These include expenses not directly linked to the department where administration takes place but attributable to the broader facility and allocated proportionally to the cost center based on the activity provided. Such costs include intermediate healthcare services, shared services, general and administrative expenses, depreciation, and non-healthcare goods and services. To this end, 25% was added to the intermediate calculation thus obtained, based on the general assumption that these structural costs represent approximately 20% of the total cost of a healthcare service [12].

#### Patient perspective

The cost areas investigated in the “EASIER for you 2?” questionnaire were categorized into non-healthcare direct costs and indirect costs.

Non-healthcare direct costs refer to out-of-pocket resources the patient must spend to access treatment and included transportation costs to/from the MS center, valued based on a per-kilometer rate specific to the mode of transport declared by the patient [14, 16–18] and expenses for formal assistance, such as accompaniment to the center, care for family members (e.g., babysitter), or household help (e.g., cleaning services) during the time the patient is at the MS center [19].

Indirect costs represent the economic value of productivity loss experienced by the patient due to reduced functioning or by the caregiver providing informal support and pertained loss of patient productivity, calculated based on the number of hours the patient was unable to perform daily work or non-work-related activities [19, 20] and informal care, expressed as the time spent by an informal caregiver providing assistance or accompanying the patient to the MS center [19, 20].

#### Societal perspective

The societal perspective encompasses all costs included in the two previously described perspectives, including direct healthcare and non-healthcare costs, as well as indirect costs.

## Results

### Part 1: time and resources for SC administration

The final database included 274 SC natalizumab administration procedures across the 14 participating centers. The number of procedures evaluated per center ranged from 4 to 40. Of these, 9 procedures (3%) were excluded from the analysis due to missing measurements that prevented the estimation of the intended outcomes. Therefore, a total of 265 procedures were included in the analysis. Patients had undergone a mean of 62 previous IV administrations (standard deviation or SD = 42; median = 52; range = 0–200) and 4 previous SC administrations (SD = 3; median = 4; range = 0–17).

Table 1 shows that patients spent on average less than half an hour (mean = 26.6 min; SD = 23.1 min; median = 20.3 min; interquartile range or IQR = 11–35 min) in the MS center to receive the SC administration of natalizumab, of which about 2.5 min were dedicated to the actual administration.

**Table 1:** Patient and HCP time spent for the subcutaneous administration

	Patient time (min)		HCP time (min)	
	Mean (95% CI)	Proportion (%)	Mean (95% CI)	Proportion (%)
Pre-administration^a^	8.7 (7.6–9.9)	33%	5.1 (4.4–5.9)	38%
Administration	2.4 (2.2–2.6)	9%	2.3 (2.2–2.5)	17%
Post-administration^b^	15.5 (13.0–18.0)	58%	6.2 (5.4–7.0)	46%
Total	26.6 (24.3–30.2)	100%	13.5 (12.1–14.8)	100%

In some cases, rounding prevents the sum to seem precise

^a^Including tasks 1 to 4 of Online Resource 1

^b^Including tasks 6 to 9 of Online Resource 1

*CI* Confidence interval; *HCP* Healthcare professionals, *min* Minutes, *SD* Standard deviation

On average, HCPs spent around 14 min (95% CI 12.1–14.8) of active working time administering SC natalizumab, of which roughly 5 min (95% CI 4.4–5.9) were allocated to preparation prior to the actual administration and 6 min were dedicated to post-administration work (95% CI 5.4–7.0) (Table 1).

The mean chair time per administration was 27.3 min (95% CI 24.4–30.2), with a median of 21.4 min (IQR 11–35) and 95% CI of mean value of 24.4 to 30.2.

#### Regression analysis on the 14 centers (EASIER 2 data only)

The time estimates (patient/HCP/chair) obtained from the random-intercept regression model adjusted for the number of previous SC and IV administrations are shown in Table 2. The results estimated with the regression analysis are aligned with data observed and described previously.

**Table 2:** Patient and HCP times estimated by the regression model for SC administration in all the 14 centers

	Patient time (min)		HCP time (min)	
	Mean	95% CI	Mean	95% CI
Pre-administration^a^	10.8	6.8 to 14.7	6.2	3.4 to 8.9
Administration	2.6	1.9 to 3.3	2.6	1.9 to 3.3
Post-administration^b^	12.5	3.6 to 21.5	5.2	2.7 to 7.6
Total	26.1	16.0 to 36.2	14.0	9.9 to 18.1

In some cases, rounding prevents the sum to seem precise

^a^Including tasks 1 to 4 of Online Resource 1

^b^Including tasks 6 to 9 of Online Resource 1

*CI* Confidence interval; *HCP* Healthcare professionals, *SD* Standard deviation

The regression analysis indicates that center-related variability accounts for approximately 40% of the total variability (i.e., the variance of the random intercept relative to the total variance).

Mean chair time was 26.32 min (95% CI 16.24 to 36.4).

As in the previous section, 51 records were excluded from pre- and/or post-administration analyses due to validity issues. The regression model estimates were consistent with the descriptive statistics reported in the previous section.

#### Regression analysis comparison between SC (EASIER 2 data) and IV (EASIER data)

Eight centers participated in both the EASIER [13] and EASIER 2 studies and a total of 542 procedures (367 IV procedures from the EASIER [13] study and 175 SC procedures from the EASIER 2 study) were used to estimate the time savings associated with SC administration vs IV administration. The time estimates obtained from the random-intercept regression model from the 8 centers here analyzed were consistent with those obtained when analyzing all the 14 centers, which are reported in Online Resource 2. Table 3 shows the results of the regression analysis used to estimate the time reduction, both in terms of absolute difference (delta SC vs IV) and percentage decrease.

**Table 3:** Reduction of HCP, patient, and chair time for subcutaneous administration compared to intravenous administration in the 8 centers involved in both the EASIER and the EASIER 2 studies

Estimated time (min)	Difference SC vs IV	95% CI	% Decrease	95% CI
Patient time	− 114.0	− 126.8 to − 101.2	− 77.4%	− 99 to − 61%
Pre-administration	− 7.7	− 17.9 to 2.6	− 32.7%	− 136 to 8%
Administration	− 69.6	− 73.7 to − 65.4	− 94.4%	− 113 to − 80%
Post-administration	− 38.6	− 45.4 to − 31.8	− 75.3%	− 128 to − 48%
HCP time	− 8.7	− 11.6 to − 5.7	− 34.8%	− 58 to − 19%
Chair time	− 82.5	− 92.4 to − 72.7	− 74.2%	− 98 to − 56%

*CI* Confidence interval, *HCP* Healthcare professionals, *IV* Intravenous, *SC* Subcutaneous

Therefore, SC administration of natalizumab allowed for a reduction in the total time a patient spends at the MS center by nearly 2 h, a decrease in chair time by almost an hour and a half, and a reduction in active HCP time by approximately 9 min, resulting in a significant impact in terms of both organization and costs for each MS center.

### Part 2: How much EASIER 2? questionnaire. HCP preferences and impact on MS centers

Overall, 17 “How much EASIER2?” questionnaires were completed by 11 of the 14 centers enrolled in the study. Seven questionnaires were filled out by nursing staff, nine by neurologists, and one by another unspecified HCP. All respondents, regardless of their role or affiliated center, rated on a 5-point Likert scale their experience with SC natalizumab compared to the IV formulation as either “very favorable” (76.5%) or “favorable” (23.5%). In particular, 55.6% of neurologists rated it as “very favorable” and 44.4% “favorable”, whereas 100% of the remainder HCPs as “very favorable”.

Regarding the organization of MS centers about SC natalizumab administration, only one HCP reported that SC natalizumab administration was reimbursed through the day-hospital hospital tariff, whereas all other HCPs declared that the service was reimbursed either as a basic outpatient specialist procedure or under a complex outpatient tariff, according to the Italian National Outpatient Services Nomenclature.

Subcutaneous administration was carried out in the same rooms used for intravenous administration in 8 centers (in 7 of these, during the same hours; in one, at different times), whereas the remaining 3 centers have rooms dedicated exclusively for SC administration.

Nearly all centers reported that the availability of SC natalizumab will not negatively impact the hospital pharmacy in terms of time required for purchase planning. Specifically, 2 centers expected a slight reduction in the time the pharmacy will need to dedicate, while 7 did not anticipate any changes. Only 2 centers reported that the availability of SC natalizumab might slightly increase the time required for purchase planning.

**Fig. 1:**
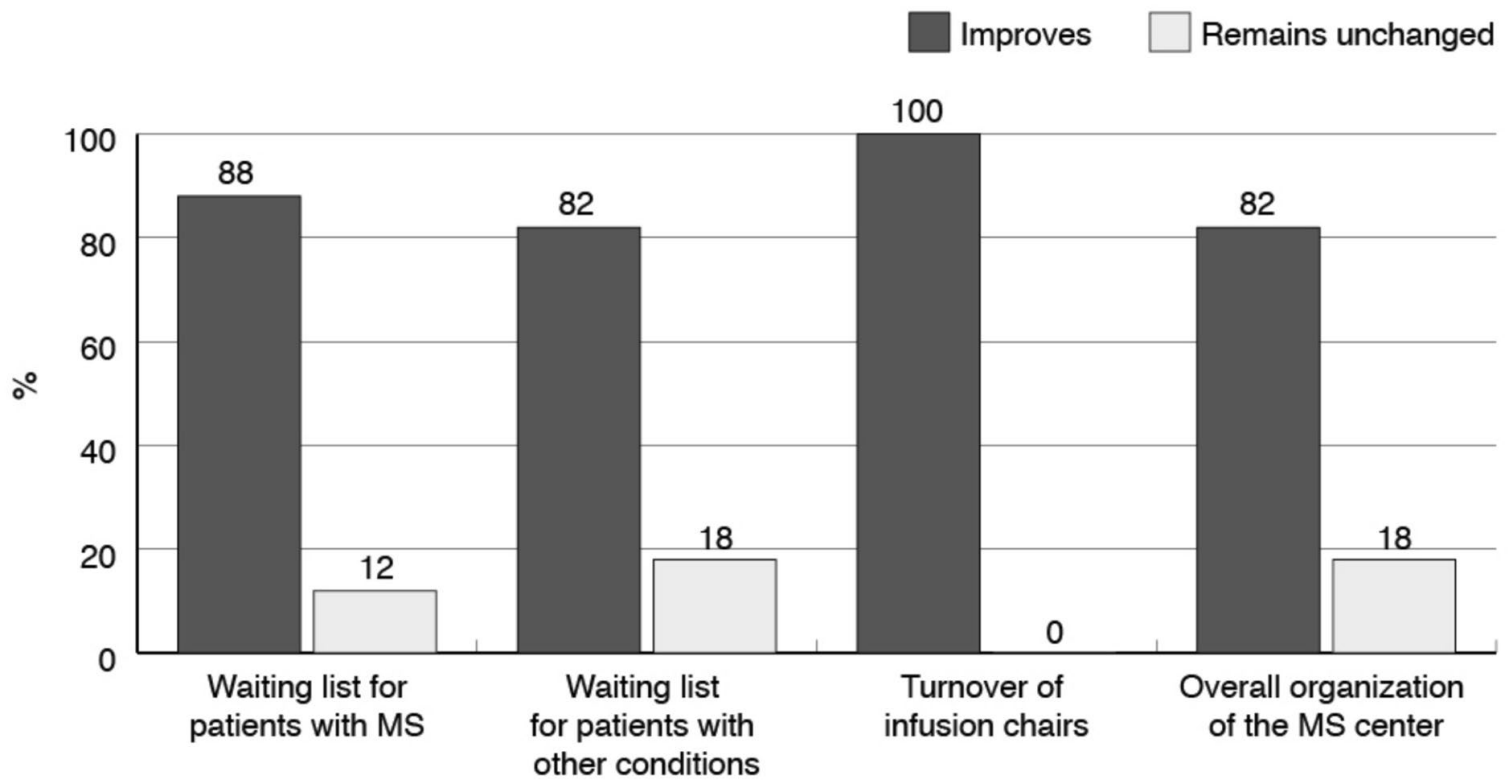
Expected or observed consequences after SC natalizumab availability according to HCP responders to "How much EASIER 2?" Questionnaire

Finally, nearly all centers agreed that the use of SC natalizumab compared to the IV formulation in clinical practice will have positive effects, including reducing patient waiting lists for MS (88%) and other conditions (82%), speeding up the turnover of infusion chairs (100%), and improving the overall organization of the MS center (82%) (Fig. 1).

### Part 3: EASIER for you 2? questionnaire. Impact on the patient

A total of 242 “EASIER for you 2?” questionnaires were correctly completed and analyzed (Table 4).

**Table 4:** Data from the responders of the survey “EASIER for you 2?”

	N. (%) responders	Mean (SD)	Range
Responders data			
Responders	242 (100%)		
Responder type	239 (99%)		
Patients	235 (98%)		
Caregivers	4 (2%)		
Age	239 (99%)	39.6 (11.1)	19–69
N. male	62 (26%)	39.3 (10.9)	19–67
N. female	177 (74%)	39.6 (11.2)	19–69
EDSS	193 (80%)	2.4 (1.9)	0–9
Satisfaction with current SC natalizumab	235 (97%)		
No	5 (2%)		
Yes	230 (98%)		
Preference about natalizumab formulation	235 (97%)		
IV	13 (6%)		
SC	201 (86%)		
No preference	21 (9%)		
Impact of SC natalizumab on everyday life			
Hours per day lost due to administration	224 (93%)	4.2 (3.0)	1–24
Among those who have a paid job	131 (57%)	3.8 (2.6)	0–8
Hours reimbursed through welfare measures	110 (45%)	63.0 (46.2)	0–100
Consequences on work activity	118 (49%)		
Permanent interruption	7 (6%)		
Reduction in working hours	11 (9%)		
Downgrade in job responsibilities	14 (12%)		
No change	86 (73%)		

*EDSS* Expanded disability status scale, *IV* Intravenous, *SC* Subcutaneous, *SD* Standard deviation

Almost all respondents were patients (98%), with a male-to-female ratio of 1:3 (26% men and 74% women). The mean age of the entire cohort was 40 years, with no differences between males and females (Table 4).

Expanded Disability Status Scale (EDSS) data were available for 80% of patients and showed a mean of 2.4 (Table 4).

About the use of previous treatments other than natalizumab, in general, a similar trend was observed across all types of medications: patients either had never taken a given formulation, or, if they had, they had been on treatment for more than 12 months. In particular, 30%, 23%, and 38% had already taken oral, intramuscular, and intravenous drug other than natalizumab, respectively, for more than 12 months. Moreover, 43% had already been treated with subcutaneous drugs for more than 12 months and 4% had a schedule providing its administration every other week. Almost 90% of the sample had been treated with IV natalizumab for a mean of 65 months and had received a mean of 4 SC natalizumab administrations. The number of previous SC natalizumab administrations was consistent with the measurements reported by HCPs in part 1 of the study. Almost all patients expressed satisfaction with their current treatment, and specifically, 86% stated a preference for the SC formulation.

**Fig. 2:**
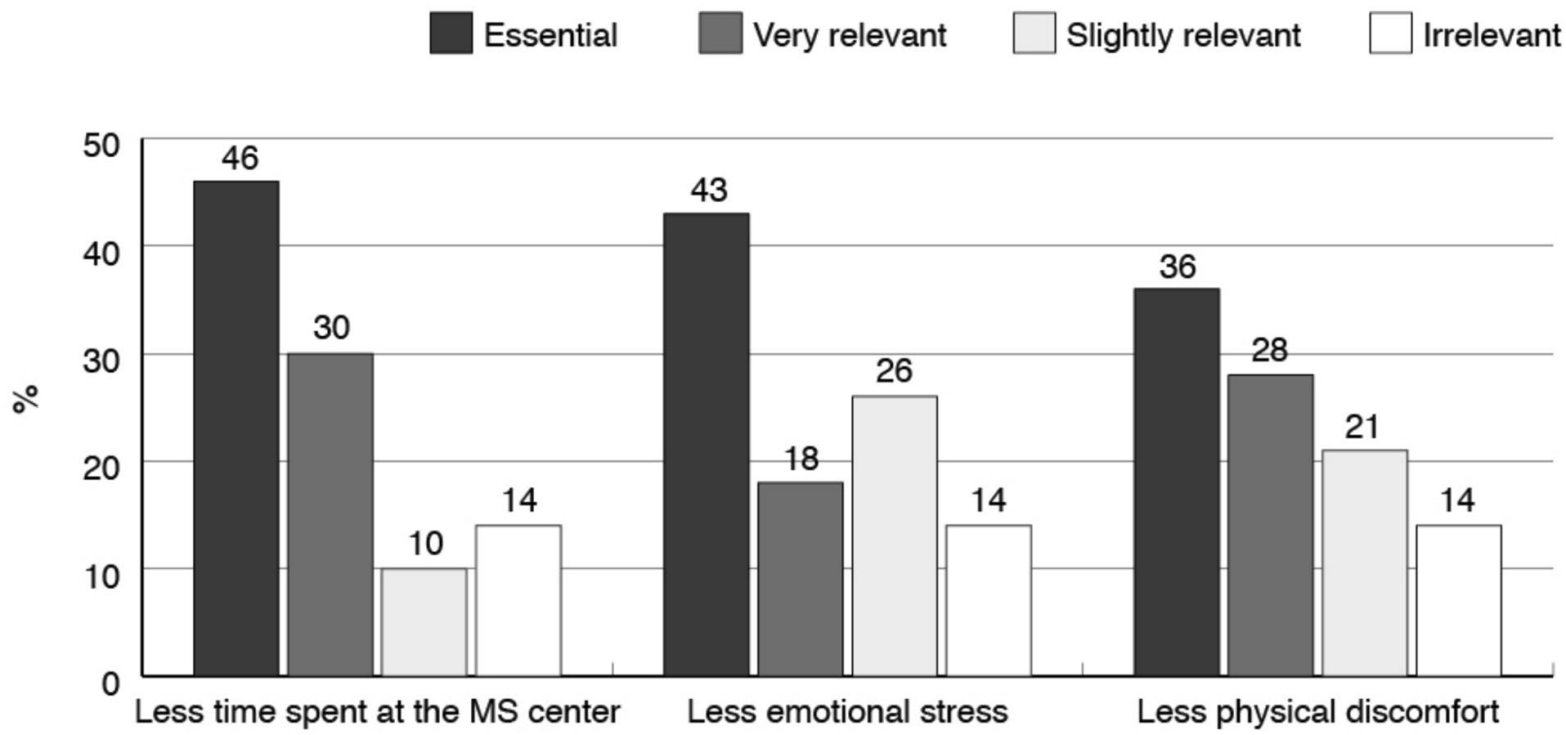
Importance of the reasons guiding patients toward preferring SC over IV administration. *MS* multiple sclerosis, *SC* subcutaneous

Patients were asked to evaluate the importance of the following reasons for preferring one formulation over another: “less time spent at the MS center”, “less emotional stress”, and “less physical discomfort”. The reason “less time spent at the MS center” was considered very relevant or essential in choosing SC administration by 77% of patients, followed by “less physical discomfort” (65% of patients), and “less emotional stress” (60% of patients) (Fig. 2).

Traveling to the MS center to receive treatment resulted in a loss of 4.2 h per day on average. It should be noticed that 57% of the sample had paid employment, and these individuals had to take a mean of 3.8 h off work to travel to the MS center and receive SC natalizumab administration (Table 4); 63% of these hours were reimbursed through welfare measures. According to patient reports, the need to periodically take time off to visit the MS center for treatment did not affect work activity in 73% of cases (Table 4). Conversely, it led to job interruption in 6% of the sample, a reduction in working hours (either self-imposed or employer-imposed) in 9%, and a downgrade in job responsibilities in a further 12% of patients (Table 4).

The round trip to the MS center covered a mean distance of approximately 70 km, mostly traveled by private transport, with a mean duration of about one hour and a half (Online Resource 3). The distance traveled using public transport was shorter (45 km), but, as expected, took longer (almost 2 h).

Only 7 patients (3.3%) reported using both private and public transport to reach the MS center, covering an average total distance of 80 km and a total travel time of 133 min.

There was considerable variability in both the distance patients traveled to reach the MS center and the time required to do so (Online Resource 3).

About half of the patients (51%) were accompanied to the MS center. Specifically, almost all those patients (98%) were accompanied by at least one informal caregiver (81% by one person, 15% by two, and 2% by three), whereas only one patient (1%) was accompanied by a paid caregiver. Approximately 50% of the accompanying persons were between 40 and 60 years old, 72% were men, and more than half (55%) had paid employment.

Only 9 patients (4%) declared that they used paid personnel to replace them at home during the time they were at the MS center to receive treatment with natalizumab: 2 patients employed a babysitter, 1 patient needed a housekeeper, and 6 patients did not specify the type of professional role.

#### Quality of life

The mean perceived QoL on a type of day without natalizumab administration was 74.8 on a 0–100 VAS scale (median 80, range 5–100) (Table 5 and Online Resources 4 and 5).

**Table 5:** Quality-of-life assessment of a day based on the type of administration (scale 0–100)

Type of day	Full cohort mean (SD)	Patients with EDSS < 4 mean (SD)	Patients with EDSS ≥ 4 mean (SD)
Without natalizumab administration	74.8 (19.8)	79.7 (17.3)	60.6 (18.8)
With IV administration	56.9 (24.8)	58.8 (22.5)	50.9 (25.4)
With SC administration	70.4 (22.0)	70.4 (20.9)	61.9 (23.2)

*EDSS* Expanded disability status scale, *IV* Intravenous, *SC* Subcutaneous, *SD* Standard deviation

On days when IV administration was received, the QoL dropped to 56.9 (median 60, range 0–100), whereas on days when SC administration was received, the QoL was 70.4 (median 75, range 0–100), slightly lower than on days without administration (Table 5 and Online Resource 4).

On average, the QoL of patients with an EDSS score < 4 was higher than that of patients with an EDSS score ≥ 4 in every type of day. In patients with EDSS score ≥ 4, the QoL decreased on days with IV administration, whereas was comparable on days without administration and with SC natalizumab administration (Table 5 and Online Resource 4).

The QoL values reported for the three types of days were compared using a linear regression model (Online Resource 4). In a scenario analysis, the model was also adjusted for EDSS score (Table 6).

**Table 6:** Results from the linear regression model

Type of day	No EDSS		Continuous EDSS		EDSS ≥ 4 vs EDSS < 4	
	Delta	95% CI	Delta	95% CI	Delta	95% CI
With IV administration vs without administration	− 18.57	− 21.62 to − 15.52	− 18.24	− 21.41 to − 15.07	− 18.25	− 21.42 to − 15.08
With SC administration vs without administration	− 5.34	− 8.39 to − 2.29	− 6.97	− 10.12 to − 3.82	− 6.94	− 10.10 to − 3.80
With SC vs IV administration	13.23	10.15 to 16.31	11.27	8.08 to 14.45	11.27	8.08 to 14.45
Age (years)	− 0.04	− 0.27 to 0.18	0.20	− 0.07 to 0.47	0.12	− 0.14 to 0.39
Gender (M vs F)	2.65	− 3.17 to 8.42	4.53	− 1.68 to 10.74	4.09	− 2.24 to 10.43
Previous IV administration (yes vs no)	1.60	− 7.64 to 10.84	7.41	− 2.81 to 17.62	6.86	− 3.55 to 17.28
Previous SC administration (yes vs no)	4.06	− 6.15 to 14.26	0.76	− 9.57 to 11.08	0.08	− 10.69 to 10.53
Paid work (yes vs no)	4.11	− 0.89 to 9.12	0.41	− 5.01 to 5.83	0.45	− 5.16 to 6.05
EDSS			− 3.34	− 4.87 to − 1.81	− 13.20	− 20.56 to − 5.85

*CI* confidence interval, *EDSS* Expanded Disability Status Scale,* F* female, *IV* intravenous, *M* male, *SC* subcutaneous

The EDSS variable was included both as a continuous parameter and as a categorical parameter (patients with a score ≥ 4 versus patients with a score < 4).

The decrease in QoL perceived by patients on days when they received SC natalizumab compared to days when they did not receive treatment was significantly smaller than the reduction perceived on days when they received IV natalizumab (− 5.34 vs − 18.57). SC administration was associated with an improvement in perceived QoL of approximately 13 points compared to IV administration (95% CI 10.15–16.3). Adjustment for the EDSS variable confirmed the base case results (Table 6). In particular, the EDSS score was strongly correlated with QoL, both when considered as a continuous variable and as a dichotomous variable. In fact, each 1-point increase in the EDSS score was associated with a decrease in QoL of − 3.34 points (95% CI − 4.87 to − 1.81). Moreover, patients with an EDSS score ≥ 4 had a QoL that was 13.20 points lower (95% CI − 20.56 to − 5.85) compared to patients with an EDSS score < 4.

Finally, a possible interaction between disease severity and the type of day on which QoL was assessed was tested.

The reduction in QoL on days with natalizumab administration compared to non-administration days, as perceived by more severely affected patients, was smaller than that reported by less severely affected patients (interaction test *p* = 0.003). However, this effect was not observed when comparing the two types of administration: in fact, QoL on the day of SC administration was higher than on the day of IV administration, regardless of the severity of the patients (interaction test *p* = 0.929).

**Fig. 3:**
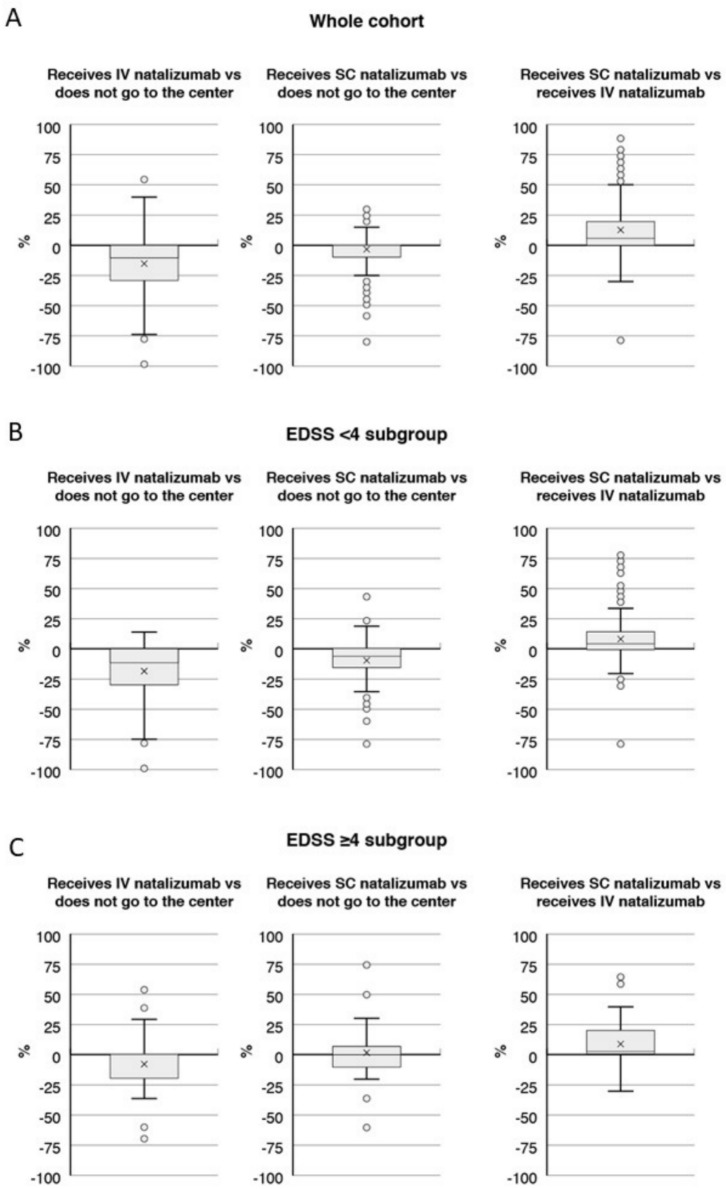
Box-and-whisker plots of the difference in quality of life measured on a type of day when the patient goes to the center to receive IV natalizumab compared to when they do not go to the center (left), when the patient goes to the center to receive SC natalizumab compared to when they do not go to the center (center), and when the patient goes to the center to receive SC natalizumab compared to when they go to the center to receive IV natalizumab (right). The width of the box represents the interquartile range, the horizontal line within the box represents the median, the mean value is indicated with a cross, and the length of the whiskers represents the range that includes approximately 95% of the observations. **A**: full cohort; **B**: EDSS < 4 subgroup; **C**: EDSS ≥ 4 subgroup

Figure 3 shows the distributions of changes in QoL and the comparison within subgroups with EDSS < 4 and ≥ 4.

### Economic impact analysis

#### MS center perspective

Table 7 shows the estimates of direct healthcare costs borne by the MS center for each hospital-based SC natalizumab administration procedure.

**Table 7:** Mean cost per administration procedure from the perspective of MS center (14 MS centers were considered)

Cost item	Mean (€)	95% CI
HCP time	6.09	5.50 to 6.68
Chair time	0.29	0.25 to 0.33
Materials^§^	3.30	3.03 to 3.57
Hospital costs	3.25	2.97 to 3.53
Total costs	12.93	11.99 to 13.87

^§^Including bend-aids, disposable gloves, gauze compresses, disinfectants, syringes with needle

*CI* Confidence interval, *HCP* healthcare professionals, *MS* multiple sclerosis

The total cost, including the cost of HCP time, chair usage, materials, and facility overheads, amounted to approximately € 13 per procedure.

The costs for SC and IV procedures were also compared across the 8 centers that participated in both the EASIER study [13] and the EASIER 2 study. Then, we performed a regression analysis to estimate the cost reduction, both in absolute terms (delta SC vs IV) and as a percentage reduction (Table 8).

**Table 8:** Reduction in SC vs IV administration costs (8 MS centers were considered)

Mean* time (min)	Delta SC vs IV (€)	95% CI	% Reduction	95% CI
HCP time	− 12.06	− 13.83 to − 10.29	− 66.2%	− 91 to − 49%
Chair time	− 0.34	− 0.4 to − 0.28	− 56.2%	− 78 to − 40%
Materials	− 3.08	− 3.31 to − 2.84	− 44.6%	− 53 to − 38%
Hospital costs	− 2.21	− 2.68 to − 1.73	− 35.8%	− 50 to − 25%
Total costs	− 20.46	− 22.73 to − 18.2	− 64.2%	− 81 to − 51%

*Mean was estimated from the regression model

*CI* confidence interval, *HCP* healthcare professionals, *IV* intravenous, *SC* subcutaneous

Compared to IV administration, SC administration resulted in a savings of nearly € 20 per procedure considering the MS center perspective.

#### Patient and societal perspective (data from 14 MS centers were considered)

About non-healthcare costs, the transportation expenses borne by the patient to reach the MS center amounted to a mean of approximately € 32, ranging from € 0.32 to € 363 (Online Resource 6). In addition, among patients who reported using formal assistance for themselves, their relatives, or for household management during travel and stay at the MS center, 9% relied on a professional caregiver. For them, this resulted in an expense of € 33.41 per administration, which corresponds to an average of € 1.24 across the entire patient population (Online Resource 6).

The time spent traveling from home to the MS center and staying there to receive a subcutaneous administration of natalizumab represented an indirect cost to society, as the patient was unable to carry out their usual activities—whether formally paid or not—during this period. From a financial perspective, three sub-indicators were developed: (1) the cost borne by the individual due to lost earnings, (2) the cost borne by society through social security systems, and (3) the cost borne by society to replace the patient in their various active roles, such as a parent caring for children, an adult assisting elderly parents, or someone engaged in domestic tasks or volunteering.

This analysis found that the time involved—travel + waiting + SC administration—resulted in a mean loss of 3.8 working hours. This time was reimbursed by social security institutions at an average rate of 63%.

The indirect costs of IV administration were estimated by considering only the cost differences due to the reduced time required for natalizumab administration, in order to avoid bias caused by possible differences in the composition of the patient population enrolled in the EASIER study [13] compared to that enrolled in the present study. Details of these indirect costs are shown in Online Resource 7. On average, the total per administration amounted to approximately € 29 in social costs and € 12 in direct financial burden to the patient, for a total cost to society of € 41. Overall, 51% of patients reported receiving assistance from informal caregivers for transportation to the MS center (an average of 1.2 caregivers per administration). Based on the mean number of hours lost as reported by patients, this resulted in a societal cost (averaged across all patients) of € 48.77 per accompanied administration (SD € 79.15; median € 10.69; range € 0–558). In the absence of specific data, the cost of informal care was adjusted by assuming the same caregiver frequency as in the EASIER study [13] (65%).

This resulted in a societal cost (averaged across all patients) of € 31.64 per administration (SD € 51.46; median € 6.37; range € 0–363).

#### Total cost across the three perspectives considered (data from 8 MS centers were considered)

In conclusion, SC natalizumab entailed a mean cost per administration of € 13.01 from the MS center perspective, € 45.03 from the patient’s perspective, and € 118.44 from the societal perspective (Table 9).

**Table 9:** Direct healthcare costs, direct non-healthcare costs, and indirect costs per administration procedure (8 MS centers were considered)

Cost type	MS center perspective (€)	Patient perspective (€)	Societal perspective (€)
Direct healthcare costs	13.01		13.01
Direct non-healthcare costs		33.23	33.23
Transport		31.98	31.98
Formal assistance		1.24	1.24
Indirect costs		11.81	72.21
Work time lost		11.81	34.13
Unpaid activities			6.43
Informal care			31.64
Total SC administration	13.01	45.03	118.44
Total IV administration (elaborated by EASIER study)	32.67	52.05	177.62
Delta SC vs IV	−19.66 (−60.2%)	− 7.02 (− 13.5%)	− 59.18 (− 33.3%)

*IV* intravenous, *MS* multiple sclerosis, *SC* subcutaneous

The findings of this analysis indicate that, in comparison to IV administration, SC administration yields savings of € 19.66 from the MS center perspective, € 7.02 from the patient’s perspective, and € 59.18 from the societal perspective (Table 9) for a single administration.

## Discussion

The EASIER 2 study was designed to evaluate the impact for MS centers, patients, and society of using SC natalizumab compared to IV natalizumab in the Italian healthcare setting. The study confirms the substantial benefits of SC over IV administration of natalizumab in patients with RRMS in terms of time, costs, QoL, preference, and MS center organization. In fact, the use of SC administration of natalizumab, compared to the IV one, reduced the consumption of healthcare and non-healthcare resources, decreased the working time for HCPs, reduced the productivity loss and the burden of therapy for patients, and improved the QoL for the patients.

In part 1 of the study, the measurements carried out showed that each SC administration procedure required patients to spend, on average, around 26 min at the MS center and HCPs to dedicate approximately 14 min. Furthermore, the average chair occupancy time was about 26 min.

Comparison of time data measured on SC administration of natalizumab during the EASIER 2 study with time data measured on IV administration natalizumab during the EASIER study [13] demonstrated that SC natalizumab reduced patient time at the MS center by 77%, HCP time by 35%, and chair time by 74%. This analysis was conducted on data collected from the 8 MS centers that participated in both the EASIER 2 and the EASIER studies to make a comparison in the most reliable manner. It is important to note, however, that the regression analyses conducted in part 1 of the study on the data measured by the 14 centers participating in the EASIER 2 study and the subgroup of 8 centers participating in both studies (EASIER and EASIER 2) showed that the data were highly consistent across the different analyses. These results confirmed the robustness and reliability of the estimates across different study methodologies and different subgroups of MS centers.

In part 2 of the study, HCPs unanimously reported a favorable view of SC administration, with 76.5% rating it as “very favorable” and 23.5% as “favorable”. Among these, 100% of nurses judged the experience with the SC formulation of natalizumab to be “very favorable” compared to the IV formulation.

HCPs believed or already observed that the adoption of SC formulation instead of IV is able to speed up the turnover of infusion chairs, thereby reducing not only patient waiting lists for MS, but also from other conditions requiring intravenous infusions. Therefore, HCPs agreed that the overall management of the MS center was simplified with a positive impact on the organization and efficiency of the MS center.

The increase in patient turnover on infusion chairs with consequent improvement in waiting lists is determined not only by the reduction in the administration time of SC vs IV but also by the fact that the subcutaneous formulation allows administration outside the infusion room. In fact, in some of the centers that participated in the study, the HCP reported, via the questionnaire, that rooms dedicated to the administration of SC were organized and set up at the same times or at different times from those for IV administration.

Furthermore, especially in smaller centers where there is no infusion room dedicated to MS, SC administration can allow for easier reorganization of appointments when they need to be changed, facilitating the relocation of patients.

Also, the reduction in the active working time of HCPs toward a single patient determines the management of a greater number of patients and a possible reallocation to other activities with consequent improvement in the organization of the MS center.

Finally, the reduced consumption of consumables such as infusion sets and disposable gloves, in addition to leading to a reduction in costs (about 45%), also has the advantage of reducing the quantity of waste and, above all, disposal times, thus freeing up additional time for healthcare workers.

In part 3 of the study, patients were required to complete a questionnaire. Almost 90% of the sample treated with SC natalizumab were familiar with the drug, as they had already been treated with IV natalizumab for a mean of more than 5 years. Moreover, 98% were satisfied with SC natalizumab and 86% of patients expressed a preference for SC natalizumab. Only 6% favored the IV formulation and 9% reported being indifferent. The main reason for the preference for the SC formulation was “less time spent at the MS center”, further corroborating the important difference in patient time relieved in part 1. However, patients’ reasons regarded also advantages for SC formulations in the perceived physical discomfort and emotional stress. When assessing the overall burden experienced by patients, it is important to consider not only the time spent at the MS center but also the 4.2 h on average required for travel. Given that the majority of patients had a paid job, it is notable that 37% of the hours taken off work were not covered by welfare measures. Furthermore, in 27% of cases, the need for time off directly impacted work activities, leading to job interruption, part-time employment, or a downgrade in job responsibilities. This is particularly noteworthy, considering that the mean age of responders was under 40 years. Additionally, the impact on work activities extended beyond the patients themselves. More than half of the patients required an accompanying person, who in most cases also had a paid job, further amplifying the socio-economic burden. The economic impact of the need for paid personnel during natalizumab administration was reported in only 4% of the sample, who indicated the need for babysitters or housekeepers during treatment sessions.

The above-mentioned issues may take on a different weight for patients treated with SC vs IV natalizumab in light of the recent approval by the European Medicines Agency (EMA) [21] of self-administration, which will allow the use of SC natalizumab at home. Self-administration allows patients to manage their treatment with greater independence, reducing productivity loss, not only for themselves but also for their caregivers. Additionally, it decreases the need for external support services, such as babysitters and housekeepers, and associated direct non-healthcare costs, while allowing patients and families to better prioritize their personal life. Therefore, self-administration can make a meaningful difference for people living with MS by empowering them with greater freedom and flexibility in managing their condition, while reducing logistical challenges.

The patients participating in the study reported a perceived average QoL of 74.8 on a standard day (without natalizumab administration), as measured by a VAS with a possible score between 0 and 100.

Using the same scale, patients rated their QoL on treatment days as 70.4 on average with SC vs 56.9 on average with IV administration. Even though the mean QoL registered a decrease in days with natalizumab administration, the difference is much more evident in days with IV infusion in comparison with days with SC administration, indicating more than 13-point advantage with SC administration. These data were also confirmed by the regression analysis, which adjusted for some variables such as age, gender, previous experience with natalizumab, EDSS, and employment status. As expected, the EDSS score was strongly correlated with QoL: the greater the disability level, the lower the QoL. However, the regression analysis showed that the QoL on the day of SC administration was higher than on the day of IV administration, regardless of the disability level.

The economic analysis conducted using the collected data during the parts 1 and 3 of the EASIER 2 study shows that the average total healthcare costs of adopting SC instead of IV administration in the perspective of MS center may be reduced by almost two-thirds (− 60%). Considering the society perspective, including direct non-healthcare costs, indirect costs as well as direct healthcare costs, the current average cost per single administration can be reduced by approximately 33%. From the patient’s perspective, while transportation expenses to the MS center remain constant, a cost reduction of approximately 14% has been estimated due to the decreased time at the health facility. This results in reduced productivity losses, fewer hours required for informal caregiving by relatives and friends, and lower formal care expenditures.

Taken together, these data suggest that SC administration improves patient care by enhancing time flexibility and QoL with respect to IV infusion. Patients save a considerable amount of time (over two hours per session), enabling greater presence with family and better integration of treatment into everyday life—two factors that patients consistently rank as top priorities.

Basing on their clinical experience with patients receiving SC natalizumab, the clinicians report improved satisfaction experienced by HCPs consequent to the greater patients’ well-being.

In parallel, SC administration significantly frees up infusion chairs—with a − 82.5 min reduction in chair time—leading to improved patient turnover and organizational efficiency at MS centers, as observed by clinicians involved in this study. As confirmed by 88% and 82% of HCPs, respectively, SC natalizumab helps reduce waiting lists for both MS and other infusion-based therapies.

Another key advantage, albeit not directly measured in this study, is the greater scheduling flexibility offered by the adoption of SC formulation. Its administration does not require dedicated infusion space, making it easier to reschedule when necessary and streamlining the patient’s journey within the MS center.

These findings are corroborated by recent literature. The NOVA phase IIIb crossover study [22] included 123 patients affected by RRMS eligible for natalizumab treatment. Overall, 87.8% of participants expressed a preference for SC natalizumab over IV natalizumab, also in this case mainly because it “requires less time in the clinic” (as reported by 83% of patients). Similarly, preliminary data from the prospective ongoing observational SISTER study [23] reported a trend toward patients’ preference for the natalizumab SC route over the IV route.

From the economic point of view, in line with the results of the EASIER 2 study, a recent Italian study [24] describes a cost-minimization and budget impact analysis of SC natalizumab vs IV natalizumab. The study highlights the dominance of SC natalizumab over IV natalizumab in terms of cost savings, with estimated reductions of €2,824, €1,137, and €9,170 per patient from the perspectives of MS center, patient, and society, respectively. In the first 3 years following reimbursement, estimated savings were around € 3.2 million from the perspective of MS centers and around € 10.3 million from the perspective of society.

Concerning the strengths of this study, it places the individual at the center of the analysis, offering a comprehensive evaluation that spans operational metrics, cost analyses, and patient-reported outcomes. It uses robust time-and-motion methodology, direct HCP feedback in addition to a questionnaire (“How much EASIER 2?”), and a structured patient questionnaire (“EASIER for you 2?”) to offer multidimensional insights. Particularly noteworthy is the integration of QoL metrics—seldom included in economic analyses—underscoring the real-world impact of SC natalizumab on the lives of patients and their caregivers.

The EASIER 2 study has some limitations. First, comparative analyses rely on SC data from EASIER 2 and IV data from EASIER. Although we tried to minimize the potential for bias arising from the difference in timing between the two studies by matching centers and adjusting for patient history, potential unobserved modifications in HCP familiarity with the drug and administration process, and possible changes in clinical workflows or center organization over time, may potentially have affected the magnitude of the observed differences between SC and IV administration. In line with the cross-sectional study design, QoL data were collected at a single time point to describe patient-perceived QoL across different types of days (days without natalizumab administration, days with IV natalizumab administration, and days with SC natalizumab administration). The longitudinal trajectory of QoL after the switch from IV to SC administration could be more appropriately investigated in future studies with a longitudinal design; in our analyses, however, we observed no correlation between number of previous SC administrations (as a proxy of time from IV switch) and the reported QoL benefit, a finding potentially suggesting stability of the observed QoL benefit over time. For part 3 of the study, patient-reported outcomes such as QoL and preference were collected via a self-administered questionnaire, as required by the methodology itself. Therefore, they may be subject to recall bias or social desirability bias. Indirect costs related to productivity losses or informal care were also based on patient data collected via questionnaire rather than direct measurement, which may affect the accuracy of the economic evaluation. Furthermore, while the findings may be considered robust within the Italian healthcare context, having followed the best practice guidelines, external validity may be limited, regarding economic results, in countries with different healthcare service, reimbursement models, or MS care infrastructures.

In conclusion, the EASIER 2 study demonstrates that SC natalizumab with respect to IV natalizumab is efficient in terms of healthcare and non-healthcare resources and advantageous in supporting patient autonomy, satisfaction, and quality of life. By reducing treatment burden and increasing scheduling flexibility, SC administration shows a positive impact on the organization and efficiency of the MS center. These results provide critical evidence underpinning the integration of SC natalizumab into clinical routine and contributing to a growing recognition of the importance of patient experience and flexibility in chronic disease management, thus supporting an increasingly patient-centered management of MS.

## Supplementary Information

Below is the link to the electronic supplementary material.

Supplementary file1 (PDF 181 KB)

Supplementary file2 (PDF 137 KB)

Supplementary file3 (PDF 135 KB)

Supplementary file4 (PDF 148 KB)

Supplementary file5 (PDF 289 KB)

Supplementary file6 (PDF 140 KB)

Supplementary file7 (PDF 136 KB)

## Data Availability

The datasets generated during and/or analyzed during the current study are available from the corresponding author on reasonable request.
