# Supplementary material for: Subcutaneous natalizumab administration in relapsing–remitting multiple sclerosis: results of EASIER 2 study
Source: J Neurol. 2026 Jul 10;273(8):452. doi: 10.1007/s00415-026-13977-w (PMC13354615; doi:10.1007/s00415-026-13977-w)
Supplement: Supplementary file 3 — Supplementary file3 (PDF 135 KB) [file 415_2026_13977_MOESM3_ESM.pdf]

Journal of Neurology

Subcutaneous Natalizumab Administration in Relapsing-Remitting Multiple Sclerosis: Results of EASIER 2 Study

Massimo Filippi[1], Luigi ME Grimaldi[2,3], Vincenzo Brescia Morra[4], Antonella Conte[5,6], Cinzia Cordioli [7], Rocco Totaro[8], Giacomo Lus[9], Augusto Rini[10], Fabiana Marinelli[11], Paola Valentino[12], Paola Perini[13], Girolama Alessandra Marfia[14], Mariarosaria Valente[15], Simona Malucchi[16], Chiara Zanetta[1], Lorenzo Pradelli[17], Daria Perini[18], Laura Santoni[18], on behalf of the EASIER 2 study working Group

[1]Neurology Unit, Neurorehabilitation Unit, Neurophysiology Service, and Neuroimaging Research Unit, "Vita e Salute" University and IRCCS San Raffaele Scientific Institute, Milan, Italy; [2]Neurology Unit, Multiple Sclerosis Center, Fondazione Istituto G. Giglio, Cefalù, Italy; [3]UniCamillus–Saint Camillus International University of Health Sciences, Rome, Italy; [4]Multiple Sclerosis Clinical Care and Research Center, Federico II University - Department of Neuroscience (NSRO), Naples, Italy; [5]Department of Human Neurosciences, Sapienza, University of Rome, Rome, Italy; [6]IRCCS Neuromed, Pozzilli, IS, Italy; [7]Multiple Sclerosis Center, ASST Spedali Civili di Brescia, Montichiari Hospital (Brescia), Italy; [8]Demyelinating Disease Center, Department of Neurology, San Salvatore Hospital, L'Aquila, Italy; [9]Multiple Sclerosis Center, UOC II Neurology, Department of Advanced Medical and Surgical Sciences; University of Campania "L. Vanvitelli", Naples, Italy; [10]Multiple Sclerosis Center, Division of Neurology, A. Perrino Hospital, Brindisi, Italy; [11]MS Center - Neurology unit, F. Spaziani Hospital, Frosinone (FR), Italy; [12]Department of Neurology, Magna Graecia University of Catanzaro, Catanzaro, Italy; [13]Multiple Sclerosis Centre, University Hospital of Padua, Padua, Italy; [14]Multiple Sclerosis Clinical and Research Unit, Department of Systems Medicine, Tor Vergata University, Rome, Italy; [15]Clinical Neurology, Department of Medicine (DMED) University of Udine, Udine, Italy; [16]SCDO Neurologia - CReSM, AOU San Luigi Gonzaga, Orbassano Torino; [17]AdRes, Torin, Italy; [18]Biogen Italia, Milan, Italy

Corresponding author: Massimo Filippi; filippi.massimo@hsr.it

	**N. responders (%)**	**Mean (SD)**	**Range**
Round trip distance (km traveled)	232 (96%)		
• Private vehicle	208 (90%)	68.2 (91.4)	1–720
• Public transport	26 (11%)	45.0 (51.1)	2–194
• Taxi	6 (3%)	8.8 (8.8)	3–15
Travel time (minutes)	232 (96%)		
• Private vehicle	200 (86%)	87.1 (81.2%)	1–480
• Public transport	23 (10%)	105.3 (75.0)	5–240
• Taxi	3 (1%)	3.0	

Online Resource 3. Time and means of transport for SC procedure.

SD standard deviation
