# Supplementary material for: Subcutaneous natalizumab administration in relapsing–remitting multiple sclerosis: results of EASIER 2 study
Source: J Neurol. 2026 Jul 10;273(8):452. doi: 10.1007/s00415-026-13977-w (PMC13354615; doi:10.1007/s00415-026-13977-w)
Supplement: Supplementary file 4 — Supplementary file4 (PDF 148 KB) [file 415_2026_13977_MOESM4_ESM.pdf]

Journal of Neurology

Subcutaneous Natalizumab Administration in Relapsing-Remitting Multiple Sclerosis: Results of EASIER 2 Study

Massimo Filippi[1], Luigi ME Grimaldi[2,3], Vincenzo Brescia Morra[4], Antonella Conte[5,6], Cinzia Cordioli [7], Rocco Totaro[8], Giacomo Lus[9], Augusto Rini[10], Fabiana Marinelli[11], Paola Valentino[12], Paola Perini[13], Girolama Alessandra Marfia[14], Mariarosaria Valente[15], Simona Malucchi[16], Chiara Zanetta[1], Lorenzo Pradelli[17], Daria Perini[18], Laura Santoni[18], on behalf of the EASIER 2 study working Group

[1]Neurology Unit, Neurorehabilitation Unit, Neurophysiology Service, and Neuroimaging Research Unit, "Vita e Salute" University and IRCCS San Raffaele Scientific Institute, Milan, Italy; [2]Neurology Unit, Multiple Sclerosis Center, Fondazione Istituto G. Giglio, Cefalù, Italy; [3]UniCamillus–Saint Camillus International University of Health Sciences, Rome, Italy; [4]Multiple Sclerosis Clinical Care and Research Center, Federico II University - Department of Neuroscience (NSRO), Naples, Italy; [5]Department of Human Neurosciences, Sapienza, University of Rome, Rome, Italy; [6]IRCCS Neuromed, Pozzilli, IS, Italy; [7]Multiple Sclerosis Center, ASST Spedali Civili di Brescia, Montichiari Hospital (Brescia), Italy; [8]Demyelinating Disease Center, Department of Neurology, San Salvatore Hospital, L'Aquila, Italy; [9]Multiple Sclerosis Center, UOC II Neurology, Department of Advanced Medical and Surgical Sciences; University of Campania "L. Vanvitelli", Naples, Italy; [10]Multiple Sclerosis Center, Division of Neurology, A. Perrino Hospital, Brindisi, Italy; [11]MS Center - Neurology unit, F. Spaziani Hospital, Frosinone (FR), Italy; [12]Department of Neurology, Magna Graecia University of Catanzaro, Catanzaro, Italy; [13]Multiple Sclerosis Centre, University Hospital of Padua, Padua, Italy; [14]Multiple Sclerosis Clinical and Research Unit, Department of Systems Medicine, Tor Vergata University, Rome, Italy; [15]Clinical Neurology, Department of Medicine (DMED) University of Udine, Udine, Italy; [16]SCDO Neurologia - CReSM, AOU San Luigi Gonzaga, Orbassano Torino; [17]AdRes, Torin, Italy; [18]Biogen Italia, Milan, Italy

Corresponding author: Massimo Filippi; filippi.massimo@hsr.it

Average day	**N (%)**	**Mean (SD)**	**Median (range)**
**Full cohort**			
• Without natalizumab administration	222 (92%)	74.8 (19.8)	80 (5–100)
• With IV administration	213 (88%)	56.9 (24.8)	60 (0–100)
• With SC administration	213 (88%)	70.4 (22.0)	75 (0–100)
**Patients with EDSS 0–4**			
• Without natalizumab administration	136 (56%)	79.7 (17.3)	80 (5–100)
• With IV administration	131 (54%)	58.8 (22.5)	60 (0–100)
• With SC administration	132 (55%)	70.4 (20.9)	75 (0–100)
**Patients with EDSS ≥4**			
• Without natalizumab administration	40 (17%)	60.6 (18.8)	60 (10–100)
• With IV administration	38 (16%)	50.9 (25.4)	50 (0–100)
• With SC administration	40 (17%)	61.9 (23.2)	60 (0–100)

Online Resource 4. Quality of life assessment of a day based on the type of administration (scale 0–100)

EDSS Expanded Disability Status Scale, *IV* intravenous, *SC* subcutaneous, *SD* standard deviation
