# Supplementary material for: Subcutaneous natalizumab administration in relapsing–remitting multiple sclerosis: results of EASIER 2 study
Source: J Neurol. 2026 Jul 10;273(8):452. doi: 10.1007/s00415-026-13977-w (PMC13354615; doi:10.1007/s00415-026-13977-w)
Supplement: Supplementary file 6 — Supplementary file6 (PDF 140 KB) [file 415_2026_13977_MOESM6_ESM.pdf]

Journal of Neurology

Subcutaneous Natalizumab Administration in Relapsing-Remitting Multiple Sclerosis: Results of EASIER 2 Study

Massimo Filippi[1], Luigi ME Grimaldi[2,3], Vincenzo Brescia Morra[4], Antonella Conte[5,6], Cinzia Cordioli [7], Rocco Totaro[8], Giacomo Lus[9], Augusto Rini[10], Fabiana Marinelli[11], Paola Valentino[12], Paola Perini[13], Girolama Alessandra Marfia[14], Mariarosaria Valente[15], Simona Malucchi[16], Chiara Zanetta[1], Lorenzo Pradelli[17], Daria Perini[18], Laura Santoni[18], on behalf of the EASIER 2 study working Group

[1]Neurology Unit, Neurorehabilitation Unit, Neurophysiology Service, and Neuroimaging Research Unit, "Vita e Salute" University and IRCCS San Raffaele Scientific Institute, Milan, Italy; [2]Neurology Unit, Multiple Sclerosis Center, Fondazione Istituto G. Giglio, Cefalù, Italy; [3]UniCamillus–Saint Camillus International University of Health Sciences, Rome, Italy; [4]Multiple Sclerosis Clinical Care and Research Center, Federico II University - Department of Neuroscience (NSRO), Naples, Italy; [5]Department of Human Neurosciences, Sapienza, University of Rome, Rome, Italy; [6]IRCCS Neuromed, Pozzilli, IS, Italy; [7]Multiple Sclerosis Center, ASST Spedali Civili di Brescia, Montichiari Hospital (Brescia), Italy; [8]Demyelinating Disease Center, Department of Neurology, San Salvatore Hospital, L'Aquila, Italy; [9]Multiple Sclerosis Center, UOC II Neurology, Department of Advanced Medical and Surgical Sciences; University of Campania "L. Vanvitelli", Naples, Italy; [10]Multiple Sclerosis Center, Division of Neurology, A. Perrino Hospital, Brindisi, Italy; [11]MS Center - Neurology unit, F. Spaziani Hospital, Frosinone (FR), Italy; [12]Department of Neurology, Magna Graecia University of Catanzaro, Catanzaro, Italy; [13]Multiple Sclerosis Centre, University Hospital of Padua, Padua, Italy; [14]Multiple Sclerosis Clinical and Research Unit, Department of Systems Medicine, Tor Vergata University, Rome, Italy; [15]Clinical Neurology, Department of Medicine (DMED) University of Udine, Udine, Italy; [16]SCDO Neurologia - CReSM, AOU San Luigi Gonzaga, Orbassano Torino; [17]AdRes, Torin, Italy; [18]Biogen Italia, Milan, Italy

Corresponding author: Massimo Filippi; filippi.massimo@hsr.it

Non-healthcare costs	**Mean (SD)**	**Median (range)**
**Transport cost per patient (€)**	**31.98 (44.71)**	**19.15 (0.32–363.23)**
• Private vehicle (€)	34.39 (46.09)	20.18 (0.50–363.23)
• Public transport (€)	7.25 (8.23)	6.44 (0.32–31.23)
• Taxi (€)	13.20 (13.30)	13.20 (6.20–20.20)
**Cost for formal assistance (€)**	**1.24 (7.04)**	**0.00 (0.00–57.28)**
• In patients who benefit from it only (€)	33.41 (16.79)	35.80 (7.16–57.28)

Online Resource 6. Non-healthcare costs borne by the patient

SD standard deviation
