# Supplementary material for: Subcutaneous natalizumab administration in relapsing–remitting multiple sclerosis: results of EASIER 2 study
Source: J Neurol. 2026 Jul 10;273(8):452. doi: 10.1007/s00415-026-13977-w (PMC13354615; doi:10.1007/s00415-026-13977-w)
Supplement: Supplementary file 7 — Supplementary file7 (PDF 136 KB) [file 415_2026_13977_MOESM7_ESM.pdf]

Journal of Neurology

Subcutaneous Natalizumab Administration in Relapsing-Remitting Multiple Sclerosis: Results of EASIER 2 Study

Massimo Filippi[1], Luigi ME Grimaldi[2,3], Vincenzo Brescia Morra[4], Antonella Conte[5,6], Cinzia Cordioli [7], Rocco Totaro[8], Giacomo Lus[9], Augusto Rini[10], Fabiana Marinelli[11], Paola Valentino[12], Paola Perini[13], Girolama Alessandra Marfia[14], Mariarosaria Valente[15], Simona Malucchi[16], Chiara Zanetta[1], Lorenzo Pradelli[17], Daria Perini[18], Laura Santoni[18], on behalf of the EASIER 2 study working Group

[1]Neurology Unit, Neurorehabilitation Unit, Neurophysiology Service, and Neuroimaging Research Unit, "Vita e Salute" University and IRCCS San Raffaele Scientific Institute, Milan, Italy; [2]Neurology Unit, Multiple Sclerosis Center, Fondazione Istituto G. Giglio, Cefalù, Italy; [3]UniCamillus–Saint Camillus International University of Health Sciences, Rome, Italy; [4]Multiple Sclerosis Clinical Care and Research Center, Federico II University - Department of Neuroscience (NSRO), Naples, Italy; [5]Department of Human Neurosciences, Sapienza, University of Rome, Rome, Italy; [6]IRCCS Neuromed, Pozzilli, IS, Italy; [7]Multiple Sclerosis Center, ASST Spedali Civili di Brescia, Montichiari Hospital (Brescia), Italy; [8]Demyelinating Disease Center, Department of Neurology, San Salvatore Hospital, L'Aquila, Italy; [9]Multiple Sclerosis Center, UOC II Neurology, Department of Advanced Medical and Surgical Sciences; University of Campania "L. Vanvitelli", Naples, Italy; [10]Multiple Sclerosis Center, Division of Neurology, A. Perrino Hospital, Brindisi, Italy; [11]MS Center - Neurology unit, F. Spaziani Hospital, Frosinone (FR), Italy; [12]Department of Neurology, Magna Graecia University of Catanzaro, Catanzaro, Italy; [13]Multiple Sclerosis Centre, University Hospital of Padua, Padua, Italy; [14]Multiple Sclerosis Clinical and Research Unit, Department of Systems Medicine, Tor Vergata University, Rome, Italy; [15]Clinical Neurology, Department of Medicine (DMED) University of Udine, Udine, Italy; [16]SCDO Neurologia - CReSM, AOU San Luigi Gonzaga, Orbassano Torino; [17]AdRes, Torin, Italy; [18]Biogen Italia, Milan, Italy

Corresponding author: Massimo Filippi; filippi.massimo@hsr.it

Cost of lost time	Mean (SD)	Median (range)
**Societal costs**	**28.87 (41.95)**	**12.77 (0.00-191.36)**
• Cost of lost working time	22.43 (43.38)	0.00 (0.00-190.29)
• Cost of lost time for unpaid activities	6.43 (8.26)	3.41 (0.00-61.29)
**Direct financial burden on the patient**	**11.86 (28.40)**	**0.00 (0.00-173.43)**
**Total**	**40.73 (44.82)**	**17.90 (0.64-191.36)**

Online Resource 7. Indirect costs, i.e., costs associated with the patients' productivity loss

SD standard deviation
